# Brief communication: temporal trends of chronic diseases medications prescriptions among HIV-infected patients in Belgium: a 4-year population-based study using pharmacy claims data

**DOI:** 10.1186/s12981-024-00634-3

**Published:** 2024-07-27

**Authors:** Dieudonné Ilboudo, Calypse Ngwasiri, Isabelle Savoye, Agnès Sommet, Dominique Van Beckhoven, Jean Cyr Yombi, Fati Kirakoya-Samadoulougou

**Affiliations:** 1 Centre de recherche Epidémiologie, Biostatistique et Recherche Clinique, Ecole de santé publique, Université libre de Bruxelles (U.L.B.), Bruxelles, Belgique; 2 District Sanitaire de Banfora, Direction Régionale de la Santé des Cascades, Banfora, Burkina Faso; 3 Pharmacologie Médicale, Faculté de Médecine, Université de Toulouse III, Inserm CERPOP, CHU, Toulouse, France; 4 Epidemiology of infectious diseases, Public Health and Surveillance, Sciensano, Brussels, Belgium; 5 Department of Internal Medicine, Infectious diseases and tropical medicine, AIDS References Centre, Cliniques Universitaires Saint Luc, UCLouvain, Brussels, Belgium; 6 Department of International Health, Bloomberg School of Public Health, Johns Hopkins University, Baltimore, USA

**Keywords:** Chronic diseases medications, Prescriptions, Co-medications, People living with HIV, Belgium

## Abstract

The Objective of this study was to examine change over time of prevalence of chronic diseases medications (CDM) prescriptions among People living with HIV (PLWH) in Belgium, using Pharmanet database from 2018 to 2021. We identified 13,570, 14,175, 14,588 and 14,813 PLWH in 2018, 2019, 2020 and 2021, respectively. Prescriptions of cardiovascular diseases (CVD) medications (31.7–37.2%) and antidiabetics (7.4–9.0%), increased significantly (p for trend < 0.001 for all), while the prescription of neurological and mental disorders medications (18.0–19.3%) remained stable (p for trend = 0.11) and the prescription of chronic respiratory diseases (CRD) medications decreased from 12.2 to 10.6% (p for trend < 0.001), between 2018 and 2021. It is imperative to ensure that these medications are used appropriately.

## Background

With Highly active antiretroviral therapy, people with HIV are living longer [1] and experiencing a rising burden of multimorbidity [2]. In Europe, estimates suggest that by 2030, 84% of people living with HIV (PLWH) will have at least one comorbidity, whereas 28% will have three or more comorbidities which may include cardiovascular diseases (CVD) [3].

Age-related comorbidities in people living with HIV (PLWH) arise from a complex interplay of traditional risk factors and HIV-specific factors, complicating HIV management [3]. This is particularly relevant in Belgium, with a reported high life expectancy of 82 years [4]. According to the 2021 report on the epidemiology of AIDS and HIV infection in Belgium, 17,018 HIV-infected patients were medically monitored, and older people (≥ 50 years) represented 43% of these PLWH [5]. The aging process affects persons of both male and female sex [5]. PLWH develops comorbidities ten years earlier than the general population [6]. This survival benefit is often associated with an increase in morbidity, partly due to therapeutics-associated multimorbidity [7].

Despite the increase in the prevalence of chronic diseases among PLWH, few authors have been interested in evaluating the use of chronic diseases medications (CDM) [2]. Co-administration of multiple medications in PLWH raises the risk of hospitalization, adverse drug reactions and drug-drug interactions [8]. Therefore, prioritizing comprehensive planning for updating real-world epidemiological data regarding CDM usage within the HIV population is crucial. Real-world analyses of concomitant medication prescriptions in PLWH reveal that, over the last decade, there has been an increasing change over time in antihypertensive medications in the U.S [9]. Similar patterns were observed in Spain for anti-diabetic medications [10].

In Belgium, most common non-infectious comorbidities in PLWH were arterial hypertension, diabetes and chronic kidney disease between 2014 and 2016 [11] and non-antiretroviral (non-ARV) therapeutic classes involved in drug-drugs interactions were mostly cardiovascular and central nervous system medications between 2012 and 2016 [12]. Studies analyzing prescriptions of these CDM among PLWH in Belgium are lacking. Knowledge on the change over time of CDM prescriptions would provide a better understanding of the challenges associated with managing coexisting diagnoses in PLWH. This study aimed to examine the change over time of CDM prescription among PLWH in a real-world heterogenous population in Belgium.

## Materials and methods

### Study design and data source

This was a retrospective cohort study of claims data conducted between January 1, 2018, and December 31, 2021 in Belgium (including COVID-19 period). Data were obtained from Pharmanet, a nationwide database of pharmaceutical services provided by 5,240 public pharmacies and reimbursed by compulsory health insurance [13]. The data contains information related to the identification of the prescribed medication and basic demographic characteristics of the beneficiary [13]. The database does not include information on patients’ socioeconomic, clinical and diagnosis data [13].

### Study population

Our study population consisted of patients aged 18 years and older with antiretroviral therapy (ART) prescriptions. Treatment for HIV was inferred for patients with two or three ART agents dispensed, while those with only one agent (e.g., tenofovir or lamivudine) were assumed to be treated for hepatitis B. Dispensing of tenofovir and emtricitabine was considered indicative for pre-exposure prophylaxis (PreP). These patients were excluded. Treatment characteristics and co-prescriptions were assessed from the index date until the end of data collection on December 31, 2021. The index date was the first date of ART prescription during the study period.

### Outcome definitions and measures

Chronic disease medications (CDM) prescriptions were identified based on the anatomical therapeutic chemical (ATC) codes of the prescribed medications. We categorized medicines falling under ATC codes N03, N04, N05, N06 as those used to treat neurological or mental disorders. Similarly, medications in ATC codes C01, C02, C03, C09, C10, B01 were classified as CVDs medications. Medicines under ATC codes A10 were designated as antidiabetics, and those in ATC codes R03 were considered as chronic respiratory diseases (CRD) medications.

### Baseline patient characteristics

Basic demographic characteristics available in Pharmanet database were considered: age at first ART dispensation in the current year ([18–34]; [35–49]; [50–64]; >=65 years) 2) sex (male or female); 3), and region (Flemish region, Brussels-Capital region, Walloon region). Older people were defined as those over 50 [5, 6].

### Statistical analysis

The prevalence of co-prescription of each therapeutic class was calculated and stratified by age, sex, and region of residence.

Logistic regression models were used to assess the change over time in the prescription of CDM among PLWH, adjusting for baseline demographic characteristics. The change over time of prescription was also presented by gender, adjusted for age and region. Similarly, changes over time were presented by age groups, adjusted for gender and region. The significance of regional change over time was assessed after adjusting for age and gender, with a significance threshold set at p for trend < 0.05. The analyses utilized STATA (version 17.0) and R (version 4.2.3) software.

## Results

The study population included 13,570, 14,175, 14,588 and 14,813 PLWH in 2018, 2019, 2020 and 2021, respectively. Overall, most PLWH were men (65.1%), resided in the Flanders region (44.6%), and fell in age group of 35 and 49 years (42.3%).

### Temporal trends of chronic disease medications

Overall, 44.8% vs. 16.9%, 49.9% vs. 17.6%, 50.5% vs. 17.6% and 53.0% vs. 18.8% of patients were taking at least one vs. two chronic diseases medications in addition to ARVs in 2018, 2019, 2020 and 2021, respectively. The prevalence of antidiabetic medication prescriptions increased significantly from 7.4% in 2018 to 9.0% in 2021 (*p* = 0.02). The prevalence of CVD medication prescriptions increased significantly in both sex, and across age groups 35–49 years and 50 to 64 years (*p* < 0.001). This change over time was also observed in the Flemish and Walloon regions (Table 1).

Regarding the prescriptions of CRD medications, a significant decrease was observed regardless of sex, across all age categories, and across all three regions (*p* < 0.001). Between 2018 and 2021, prescriptions for neurological and mental health disorders did not increase significantly (*p* = 0.11), and no significant change over time was observed in the sub-group analysis (Table 1).

**Table 1:** Prescriptions for the treatment of diabetes, cardiovascular diseases, chronic respiratory diseases, and neurological and mental disorders among people living with HIV over time (2018–2021)

Baselines demographics characteristics	Prevalence (%)				
	2018 (*n* = 13,570)	2019 (*n* = 14,175)	2020 (*n* = 14,588)	2021 (*n* = 14,813)	*p*-trend
**Antidiabetics**					
**Overall**	7.4	7.8	8.4	9.0	**0.002**
Sex*					
Male	7.2	7.4	8.1	8.6	**0.02**
Female	7.9	8.4	8.9	9.9	**0.03**
Age (years)**					
18–34	1.3	1.2	1.3	1.2	0.90
35–49	3.9	4.0	4.1	4.6	0.08
50–64	11.7	11.7	12.5	13.1	**0.01**
≥65	18.4	19.4	19.8	19.7	0.42
Region***					
Flemish	6.2	6.5	7.2	7.8	**0.01**
Brussels-capital	8.5	8.6	9.4	9.8	0.17
Walloon	8.5	8.9	9.4	10.2	0.14
**Cardiovascular diseases medications**					
**Overall**	31.7	33.1	34.8	37.2	**< 0.001**
Sex*					
Male	31.9	33.3	34.7	36.9	**< 0.001**
Female	31.3	32.8	34.8	37.6	**< 0.001**
Age (years)**					
18–34	6.5	7.1	6.0	6.4	0.61
35–49	18.9	19.2	20.1	22.0	**< 0.001**
50–64	47.9	48.5	49.6	51.9	**< 0.001**
≥65	72.7	75.4	76.1	75.6	0.11
Region***					
Flemish	30.0	32.1	33.9	36.6	**< 0.001**
Brussels-capital	31.8	32.4	33.7	35.5	0.11
Walloon	34.4	35.5	37.4	40.1	**0.001**
**Chronic respiratory diseases medications**					
**Overall**	12.2	11.8	10.3	10.6	**< 0.001**
Sex*					
Male	12.3	12.1	10.6	10.9	**< 0.001**
Female	11.9	11.4	9.8	10.3	**< 0.001**
Age (years)**					
18–34	8.1	7.4	6.3	6.6	**0.03**
35–49	10.8	10.5	8.8	9.4	**0.001**
50–64	13.8	14.1	12.3	12.1	**< 0.001**
≥65	19.8	15.5	14.5	14.9	**0.002**
Region***					
Flanders	12.0	11.6	9.8	10.8	**< 0.001**
Brussels-capital	10.6	10.6	9.5	9.4	**0.005**
Walonia	14.4	13.6	12.3	11.9	**< 0.001**
**Neurological and mental disorders medications**					
**Overall**	18.0	18.4	18.5	19.3	0.11
Sex*					
Male	20.1	20.2	20.4	21.3	0.17
Female	14.1	15.3	14.9	15.6	0.47
Age (years)**					
18–34	10.6	10.2	11.4	11.6	0.19
35–49	16.5	16.9	16.9	17.7	0.13
50–64	21.2	21.1	21.2	21.5	0.66
≥65	25.9	27.2	24.5	26.7	0.96
Region***					
Flemish	17.8	18.1	18.5	19.4	0.05
Brussels-capital	17.5	17.9	18.1	18.6	0.63
Walloon	19.1	19.4	19.1	19.9	0.90

*p trend for sex was adjusted for age and region; **p trend for age was adjusted for sex and region; ***p trend for region was adjusted for sex and age; bolded p-values are significant

### Aging and prescriptions prevalence of cardiometabolic diseases (CMD) medications

**Fig. 1:**
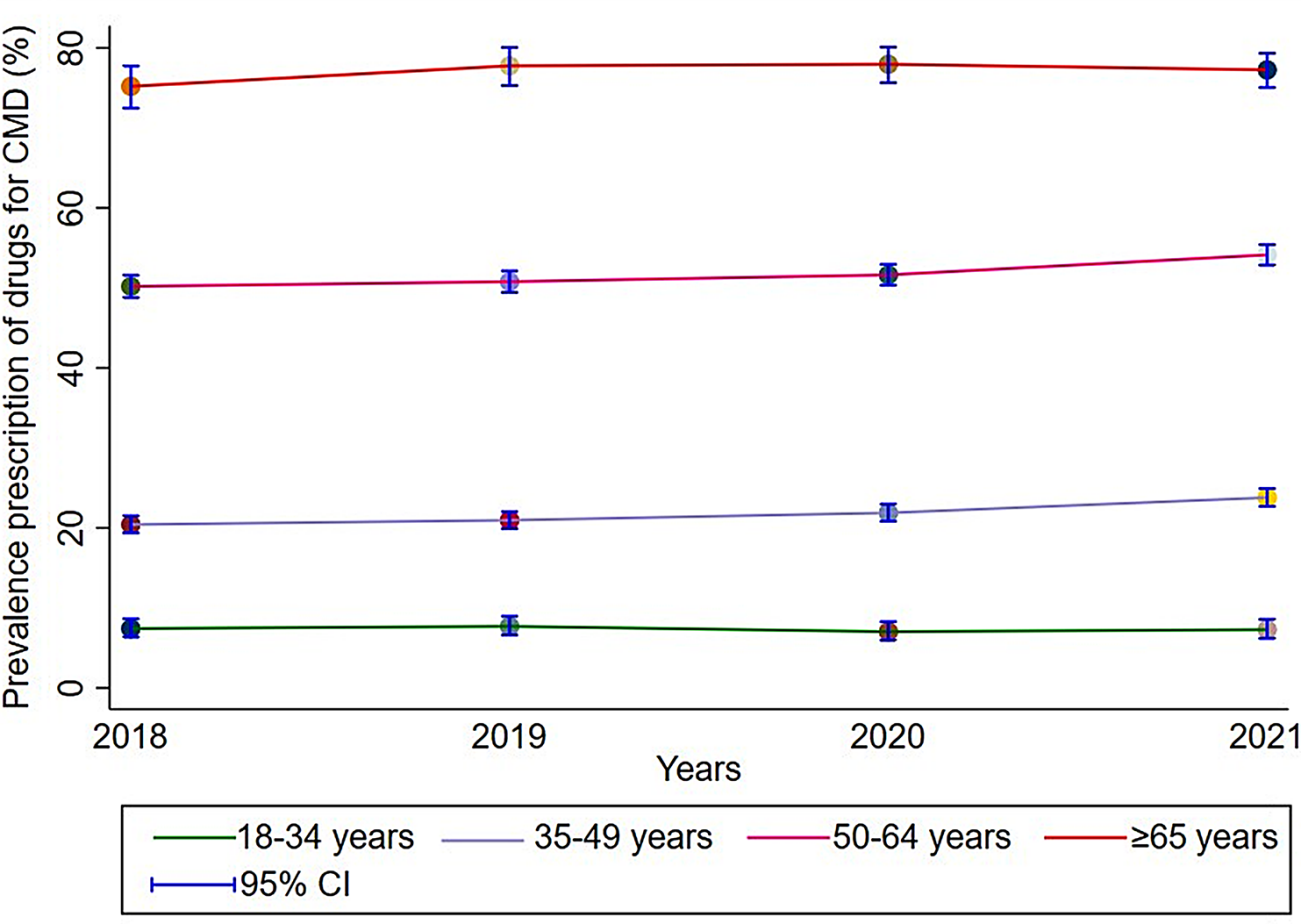
Prevalence of cardiometabolic disease medication prescriptions stratified by age among people living with HIV between 2018 and 2021 among the study population

The prevalence of cardiometabolic diseases (CMD) medication prescriptions was higher among older PLWH in Belgium compared to younger adults between 2018 and 2021. This prevalence ranged from 16.9% (CI 95%: 16.1–17.8) to 19.7% (CI 95%: 18.8–20.6) among people under 50 years and varied from 54.5% (CI 95%: 53.3–55.8) to 58.8% (CI 95%: 57.6–59.9) in PLWH older than 50 years during the study period (Fig. 1).

## Discussion

This study is the first to use the pharmacy claims database to present change over time in the prescription of CDM among PLWH in Belgium. The prevalence of CDM prescription was persistently high during the study period and was higher in subgroups of older patients (≥ 50 years). The Prevalence of antidiabetic and CVD medication prescriptions increased between 2018 and 2021 while the prevalence of CRD medication prescriptions decreased significantly during the same period.

Cardiovascular disease and diabetes were the most frequent non-infectious comorbidities among PLWH in Belgium and prevalence rates of these comorbidities increase with aging [11]. Our findings could explain the increase of the prevalence of prescription of these medications. In addition, the improvement in the management of CDM marked by the change over time of recommendations for the treatment of chronic diseases could also justify the increase in the use of medications among PLWH in Belgium. Similarly, some authors in Europe predict that 84% of PLWH will have at least one non-communicable disease and 54% of these patients will be prescribed co-medications in 2030 [3]. Among PLWH in the United Kingdom, 65% took at least one non-ARV co-medication [14].

In our cohort, prescriptions of neurological and mental disorders medications did not significantly increase between 2018 and 2021. However, it is important to note that the COVID-19 pandemic marked 2020 and 2021. Studies during this period showed an increase in mental disorders among the HIV-infected population, even though this population is more susceptible than the general population [15]. Comparatively, in Europe 54.5% of PLWH had at least one prescription of a psychotropic drug with an increasing change over time observed during 1995–2009 in a Danish cohort [16]. Older age was associated with increased drug utilization [16].

The prevalence of prescription of chronic respiratory disease (CRD) medications decreased significantly during the study period. This decline was particularly notable among those over 65 years. This decrease may be linked to the introduction of recommendations on the management of chronic lung disease and prescriptions in older people living with HIV in the European AIDS and Clinical Society (EACS) guidelines of October 2017 [17]. The implementation of these guidelines facilitated better supervision of prescriptions in the elderly and could have impacted the use of CRD medications in elderly people living with HIV in Belgium. Limited evidence exists on the use of CRD medications in Europe. However, some authors have demonstrated that the risk for CRD is significantly higher in PLWH compared to the HIV-uninfected population, although rates of lung cancer appear to be declining over the last two decades [18]. This decline could be attributed to the reduction in daily tobacco use in Belgium [19]. Notably, smoking is the leading cause of chronic obstructive pulmonary disease [20]. These findings enhance our understanding of the use of these drugs among PLWH and will contribute to guiding public health decisions in Belgium.

The principal strength of this study lies in its use of a nationwide pharmacy medico-administrative data, providing physicians and policy makers with a first-time snapshot of the change over time of chronic disease medication prescriptions among PLWH in Belgium. However, the study has limitations, such as the absence of clinical and physician-related information, hindering adjustments for external factors influencing prescription behavior.

In conclusion, this study presents a simple approach to examining change over time in chronic disease medication prescription at the national level among PLWH in Belgium. The prescribing of CDM has evolved in Belgium, likely influenced by the increasing prevalence of these co-morbidities among PLWH. These results highlight the need to continuously monitor prescriptions for chronic diseases among PLWH in Belgium. Also, ensuring appropriate use of these medications is crucial, as underuse or misuse can lead to higher morbidity and mortality rates. Further investigation into specific medication groups may reveal underlying drivers for prescription changes over time.

## Data Availability

No datasets were generated or analysed during the current study.

## Abbreviations

ART: Antiretroviral Therapy
ARV: Antiretroviral
CDM: Chronic Diseases medications
CMD: Cardiometabolic Diseases
CRD: Chronic Respiratory Diseases
CVD: Cardiovascular Diseases
EACS: European AIDS Clinical Society
HIV: Human Immunodeficiency Virus
ATC: Anatomic Therapeutic and Chemical
PLWH: People living with HIV
